# The Use of a New Passive Sampler for Ozone and Nitrogen Oxides Monitoring in Ecological Effects Research

**DOI:** 10.1100/tsw.2001.81

**Published:** 2001-09-24

**Authors:** Franco De Santis, Tuncay Dogeroglu, Sabrina Menichelli, Caterina Vazzana, Ivo Allegrini

**Affiliations:** CNR-Istituto Inquinamento Atmosferico, Area della Ricerca di Roma, Italy

**Keywords:** diffusive sampling, passive sampling, ozone, nitrogen oxides, nitrogen dioxide, air quality standards, siting criteria

## Abstract

A simple, cost-effective diffusive sampler is described that is suitable for measuring parts per billion (ppb) levels of ozone and nitrogen oxides. The diffusive sampler makes use of nitrite for ozone determination whereas for nitrogen oxides and nitrogen dioxide an active carbon tissue impregnated with sodium carbonate is used. Nitrate and nitrite, the formation of which is proportional to the pollutant concentration and sampling duration, are the two species analysed, respectively. Diffusion tubes have the advantage of being a low- cost, convenient way of mapping spatial distributions and investigating long-term trends of ozone and nitrogen oxides. The method is extremely useful for assessing long-term concentrations such as the annual mean for nitrogen oxides, as required by the Daughter Directive 1999/30/EC. Field tests to validate the method have been carried out at an urban background location with co-located passive samplers and continuous measurements of O_3_ and NO_x_ . An application in ecological effects monitoring for ozone is also presented.

### INTRODUCTION

Since the inception of the Framework Council Directive (96/62/EC)[1], the European Commission has been working on a Daughter Directive setting target values for ground level ozone for the protection of the ecosystem. At the same time the first Daughter Directive 1999/30/EC[2] has already established for $NO_x$ an annual limit value of 30 µg/m$^3$. One of the aims of the European Framework Directive is to assess ambient air quality on the basis of common methods and criteria. Under this Directive, the Member States are required to assess the spatial concentration distribution of air pollutants throughout their territory by using screening techniques and large scale surveys.

Conventional ozone measurement methods are typically based on ultraviolet absorption or a chemiluminescent reaction with ethylene gas, whereas $NO_x$ is routinely monitored with chemiluminescence monitors. These automated monitors are quite expensive (e.g., US $10,000), and obviously require electricity and temperature-controlled facilities for operation. Simpler measurement techniques for assessing air quality may offer a cost-effective alternative to conventional techniques for large-scale measurements carried out for mapping the air quality distribution as required by the Directive. In principle, passive sampling represents a perfect tool to characterise those areas where the limit values are expected to be exceeded and/or where other assessment methods are needed to comply with EC legislation. In contrast to active samplers in which air is brought into contact with a detector or collector device by means of a pump, diffusive samplers rely on diffusion to bring the pollutant into contact with a sorbent. Compared with the pump-dependent active sampling procedure, the main advantages of the method are cost effectiveness, simplicity, and the potential for large-scale measurements carried out at the same time.

Various designs of passive samplers have been developed for ozone. A diffusive measurement method based on the ozone reaction with indigo carmine has been described by Grosjean and Hisham [3]. The uptake rate of this device is determined from calibration against a standard gas mixture in the laboratory or on calibration in the field without any assurance of the constancy of the uptake rate whose variability is due largely to temperature and relative humidity changes.

Koutrakis et al.[4] developed a passive sampler based on the oxidation of nitrite to nitrate. Initial studies designed to test this device have shown its reliability but in subsequent use this sampler was found to be unacceptable during validation studies performed prior to field operations. Inconsistent sampler response, due to apparent changes in effective sampling face velocity (the velocity of the air at the mouth of the sampler), was identified as a likely source of sampling bias. To overcome this limitations, a battery-operated controlled flow sampler was proposed[5].

Various designs of passive samplers have been developed for $NO_x$. In particular, a large body of work has appeared in the literature concerning the application of passive devices to the determination of $NO_2$, whereas much less has been published on the performance of passive samplers for NO.

The most common passive system for the collection and determination of $NO_2$ in ambient air is the Palmes sampler[6,7], based on the use of triethanolamine (TEA) as an absorbing medium. Originally developed in the field of occupational hygiene, the Palmes sampler was first introduced for monitoring ambient $NO_2$ by Atkins[8] and since then it has been widely used for studies in urban and rural areas and for deployment in UK national surveys[9,10,11]. As far as the determination of NO is concerned, the idea at the basis of the few applications presented in the literature is to oxidise NO to $NO_2$ followed by collection on TEA. When $NO_2$ and $NO_x$ samplers are exposed simultaneously, NO can be calculated by difference. Palmes and Tomczyk[12] modified a Palmes tube by insertion of a microfiberglass disc containing $Na_2Cr_2O_7$ and $H_2SO_4$. A careful evaluation of the published literature shows that there are a number of conflicting results when the performance of Palmes tubes has been compared to real-time reference techniques such as chemiluminescence. For instance, overestimation of up to 40% of $NO_2$ has been reported by Campbell et al.[10] and by Gair and Penkett[13]. In contrast with these results, Campbell[9] and Atkins and Lee[11] reported accuracy within 7% and 3%, respectively.

In this paper we report on simple, inexpensive passive samplers for $O_3$, $NO_2$, and $NO_x$. The purpose of the work was to develop diffusion tubes for $O_3$, $NO_2$, and $NO_x$ to fulfill the required specifications for indicative measurements as requested in the Daughter Directives.

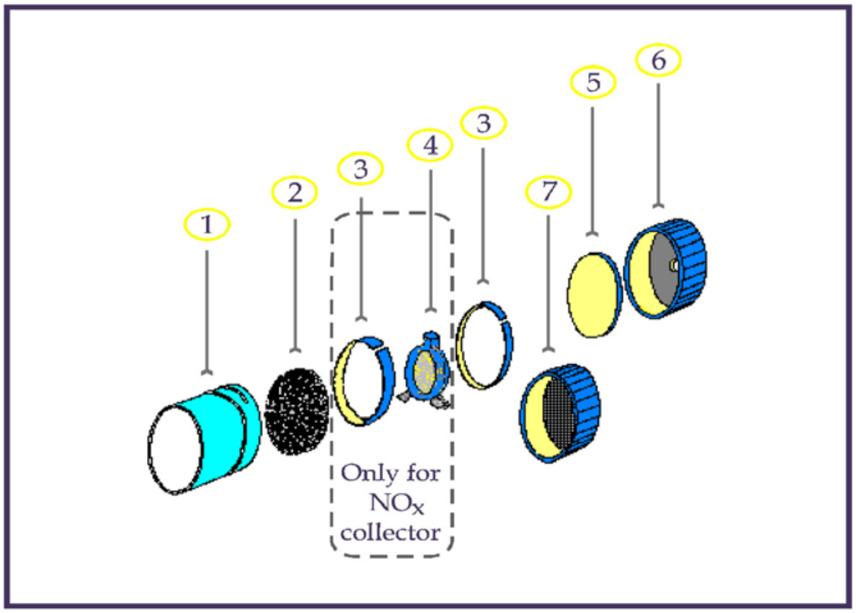

**FIGURE 1.** Exploded scheme of $NO_2/NO_x$ Diffusive Sampler: 1 – glass cylinder; 2 – oxidant, 3 – ring, 4 – absorbent; 5 – septum; 6 – plastic cap; 7 –air barrier (stainless steel screen).

## EXPERIMENTAL PART

### $NO_x$ and $NO_2$ Sampler

The sampler developed is a modification of a commercial version of a passive device for BTX (ANALYST®)[14] obtained by replacing the granular sorbent with a carbon-coated filter paper treated with appropriate reagents to trap $NO_2$ (see Fig. 1). To avoid turbulent diffusion inside the sampler, the open end was protected using a fine stainless steel screen (part 7 of Fig. 1). The carbon paper filters (Marbaglass, Rome) were coated by using a solution of 1% (w/v) sodium carbonate + 1% (w/v) glycerine in water and then dried in an oven at 70°C horizontally supported at the edges on parallel glass rods glued to a glass plate. The sampler is closed by using the septum and cap shown in Fig. 1 (parts 5 and 6) ready to be deployed in the field.

The determination of $NO_x$ is achieved by insertion of a fiberglass disk containing an aqueous solution of a suitable oxidant (a 5% aqueous solution of $CrO_3$ that oxidises NO to $NO_2$) placed at the bottom of the sampler and held in position by a stainless steel ring. On this ring is placed a polyethylene disc having three small bulges (part 3 of Fig. 1) that supports the collecting carbon paper filter on the carbon-coated filter. When $NO_2$ and $NO_x$ samplers are exposed simultaneously, NO can be calculated by the difference. After exposure, the filters were extracted in Dionex eluent (2.7 m*M* $Na_2CO_3$ and 0.3 m*M* $NaHCO_3$) and the nitrite ion concentration was determined using Ion Chromatography (Model 500, Dionex, USA equipped with a column IONPAC AS12A). The concentration of nitrite was calculated by referring to the calibration graph constructed by using the peak heights of nitrite standards. The uptake rate for $NO_2$ corresponds to the theoretical one calculated by using the Fick's law[7]. In the case of $NO_x$ the uptake is no longer a simple function of geometrical quantities and must be determined from calibration against standard gas.

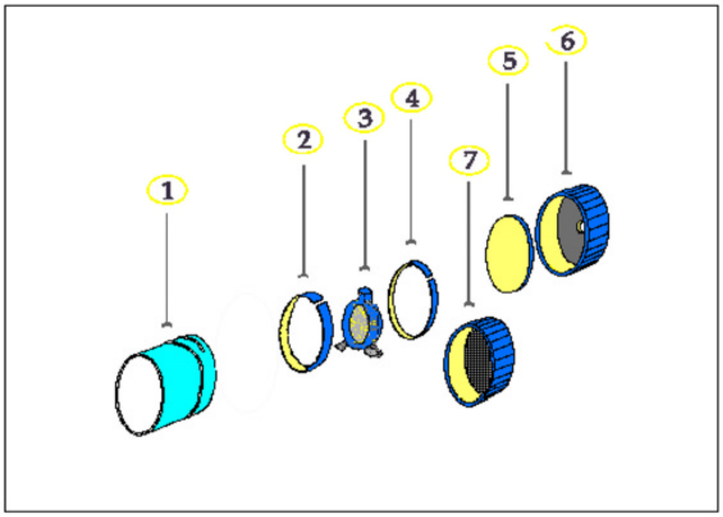

**FIGURE 2.** Exploded scheme of $O_3$ Diffusive Sampler: 1 – glass cylinder; 2 and 4 – ring; 3 – absorbent; 5 – septum; 6 – plastic cap; 7 – air barrier (stainless steel screen).

### $O_3$ Sampler

The design of the system utilised is also based on the ANALYST® design. The body of the sampler was painted black in order to shield it from light. $O_3$ is collected on a disc of a microfiber filter impregnated with an alkaline 1% solution of sodium nitrite. To avoid turbulent diffusion inside the sampler, the open end was protected using a fine stainless steel screen (part 7 of Fig. 2). Before and after sampling the screen is replaced with a polyethylene cap (parts 5 and 6 of Fig. 2). After exposure, the filters were extracted in Dionex eluent (2.7 m*M* $Na_2CO_3$ and 0.3 m*M* $NaHCO_3$) and the nitrate ion concentration was determined using Ion Chromatography (Model 500, Dionex, USA equipped with a column IONPAC AS12A). The concentration of nitrate was calculated by referring to the calibration graph constructed by using the peak heights of nitrate standards. Similarl to the $NO_x$ sampler, the uptake rate for the sampler must be determined from calibration against standard gas mixtures of ozone. Preliminary experiments carried out both in the laboratory and in the field showed that the oxidation of nitrite to nitrate is very sensitive to the light. This is in contrast to what was found by Koutakis et al.[4] and presumably is the reason for the erratic or inconsistent response previously mentioned for this type of sampler.

## RESULTS AND DISCUSSION

### Development of Passive Samplers for Ecological Monitoring

We report here on an experiment for the field validation of the passive samplers developed. Measurements from the $NO_x$ passive sampler were compared with those of a chemiluminescence analyser, the Directive reference method, by placing them near the sampling inlet of the automatic instrument. $NO_x$ passive samplers were deployed at the monitoring station of Villa Ada in Rome and the chemiluminescence analyser was calibrated daily under local quality assurance procedures. The $NO_x$ and $NO_2$ measurements made with the passive sampler were in a very good agreement with the measurements from the chemiluminescence analyser, as shown in Fig. 3. Each data point was an average of triplicates. The slopes of the linear regression lines were 1.01 and 1.02, respectively, for the two pollutants.

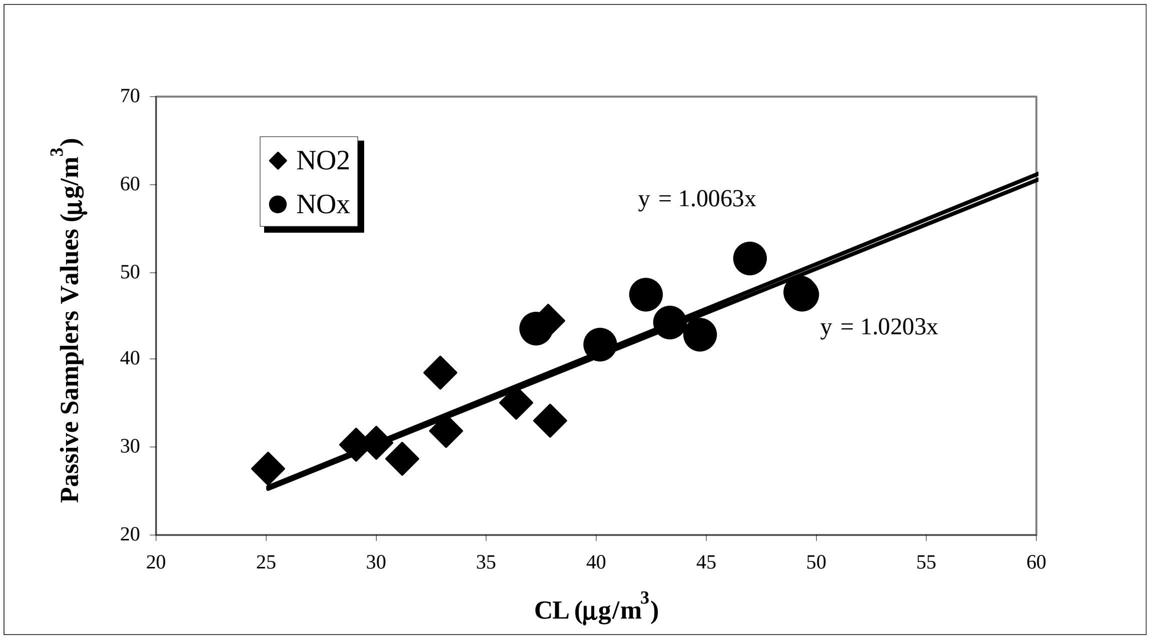

**FIGURE 3.** Comparison between Diffusive Sampler and chemiluminescence analyser for $NO_x$ and $NO_2$.

The accuracy of the passive samplers in comparison to the chemiluminescence technique expressed as percent relative error was found to be better than ±15%, with a variation coefficient of 3% for $NO_2$, 4% for $NO_x$, and 10% for NO, respectively.

To determine whether a diffusive sampler can be used for ambient monitoring it is necessary to investigate whether samplers exposed for extended periods (weeks and months) give the same integrated response as a series of short-term samplers run side-by-side. The ratio of a 4-week average concentration computed from the sum of four subsequent 1-week samples to the monthly sample can be used to indicate the self-consistency of the method. Results obtained in five subsequent tests showed a very good agreement between the monthly and the sum of the weekly samples. A ratio of $1.05 \pm 0.1$ was measured.

Measurements from the $O_3$ diffusive sampler were compared with those of an ultraviolet analyser by placing them near the sampling inlet of the automatic instrument. The samplers were also deployed at the monitoring station of Villa Ada in Rome.

The $O_3$ measurements made with the diffusive sampler were in a very good agreement with the measurements from the analyser, as shown in Fig. 4. Each data point was an average of triplicates. The slope of the linear regression line was 1.09. The ratio of a 4-week average concentration computed from the sum of four subsequent 1-week samples to the monthly sample can be used to indicate the self-consistency of the method. Results obtained in five subsequent tests showed a very good agreement between the monthly and the sum of the weekly samples. A ratio of $1.05 \pm 0.1$ was measured.

### Application to Ecological Monitoring

The potential impact of ambient ozone as a phytotoxic air pollutant on agronomic and horticultural crops has become one of the most debated issues of atmospheric pollution in recent years. The scientific basis of the recommended guidelines for ozone to protect agricultural crops

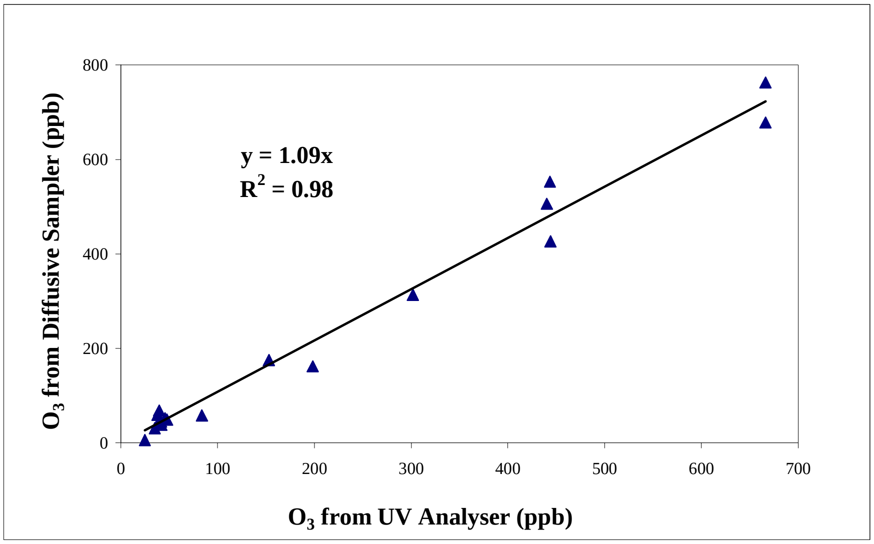

**FIGURE 4.** Comparison between Diffusive Sampler and UV Analyser for $O_3$.

comes principally from information on vegetation effects presented in the framework of the UN-ECE Long-Range Transport of Air Pollutants Convention. The current approach to pollution control measures in Europe, as adopted by the United Nations Economic Commission for Europe (UN-ECE), is to define a critical level of the pollutant. This is the highest concentration of ozone that will not cause damage to the ecosystem. The levels are specified in terms of the accumulated exposure over a threshold of 40 ppb, known as AOT40 and are measured in units of ppb.h.

For agricultural crops the AOT40 is calculated for daylight hours during 3 months, normally May, June, and July, and was set at a level of 3000 ppb.h. For forest trees the critical level is an AOT40 of 10,000 ppb.h measured for the 6- month period April to September (this is defined as the Level I approach). Although it is known that ozone (and, to a lesser extent, nitrogen oxides) can produce adverse effects on vegetation, there is not yet a clear understanding of the relationship between concentrations, duration of exposure, pollutant uptake by the plants, and adverse plant response. In fact, numerous factors can substantially affect these relationships such as soil, meteorological factors, condition of the stomata, and cellular factors which have been identified but not adequately examined in the literature. As a matter of fact, there is a lack of consensus on key aspects of an effect-cause exposure regime (for a recent review and a discussion, see De Santis[15] and Ashmore and Fuhrer[16]). In general, determining the appropriate exposure index that best links plant response to ozone depends on consideration of the biological basis for the response. To show the extent of ozone pollution it is desirable to construct maps of AOT40. However, data are limited and there is large inter-annual variation.

The ICP-Vegetation experiments have shown that the current critical level of 3000 ppb.h is regularly exceeded during the summer months in Italy and at several other European sites. However, exceedance was not always associated with a significant reduction of yield of vegetation, suggesting that other (level II) factors might be influencing the response to ozone. Initial statistical analysis indicated that proximity to local sources of $NO_x$ is an important modifying factor. Despite the potential for injurious effects, little information is available on the sensitivity to ozone of vegetation in the Italy. There are many species that have not been investigated experimentally in Italy whereas exposure response data have been obtained for a large range of crop species in the U.S., particularly through the NCLAN programme, and in other parts of Europe. A careful consideration of the experimental evidences and available information that have led to the establishment of these critical levels for ozone concentration can show that there are still many gaps and large uncertainties. In fact, viewing all the results obtained, quite a number of unresolved questions remain. In particular, in addition to the timing of exposure, effects of ozone on plants depend on environmental factors, especially factors that influence plant sensitivity via their effect on stomatal conductance. Site-specific critical levels for a particular species can be calculated by introducing weighting functions that produce scaling factors to adjust the AOT40 exceedances. A modifying factor derived from average temperature and rain data was used and the critical AOT40 values were replaced by AOT40 corrected. This leads to a reduction in the AOT40 exceedance in places with dry soils.

A simple procedure that follows Fuhrer et al.[17] was used to determine a factor (f water) from routinely measured climatological parameters that could be used to correct yield-loss estimates for wheat under limiting soil water in Italy. The factor was calculated from the relative available soil-water content determined by the balance between precipitation and potential evapotranspiration.

The correction factor was then calculated for the conditions taken from the stations of the Air Force Network spread all over Italy The results indicated that the use of a correction factor led to a significant reduction in the estimated AOT40 value, especially in the south of Italy (right part of Fig. 5).

Recently the alternative concept of Maximum Permissible Ozone Concentration (MPOC) was introduced by Gruenhage et al.[18]. This approach is a synthesis of the classical concept of critical concentration (the simple average concentration over an established period) found using passive samplers and the dose concept which is much more relevant from a toxicological point of view.

For characterising the risk to agricultural crops and other types of vegetation, average values over 24 h, 7 days, 30 days, and 90 days (all of them available by using the passive sampler developed here) can be used. Further work is underway to evaluate the utility of this approach.

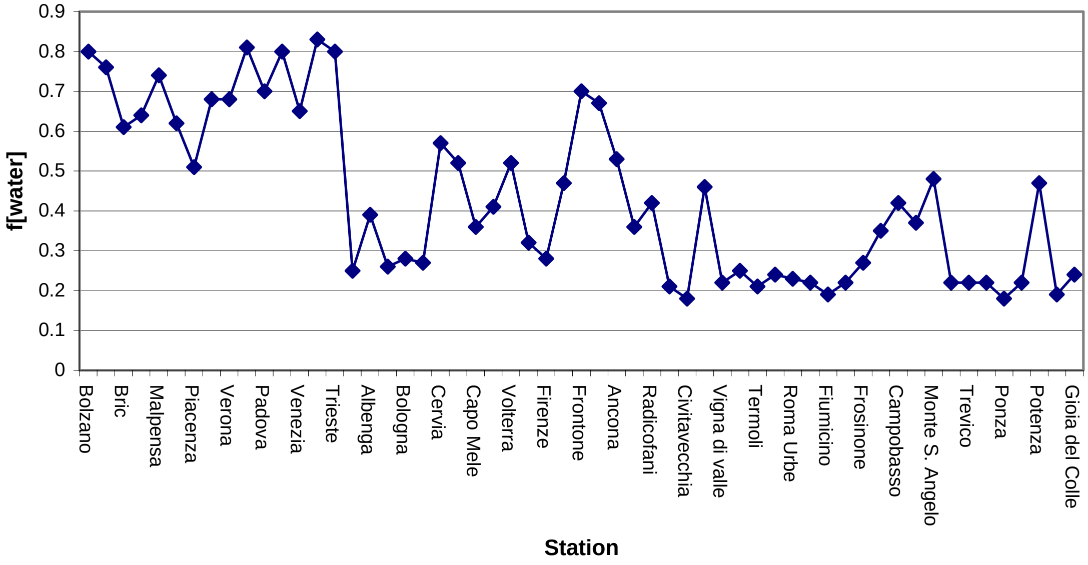

**FIGURE 5.** Calculation of the factor "f water" for 19 Italian meteorological stations.

### ACKNOWLEDGMENT

We gratefully acknowledge the assistance of the local site operators from the City of Rome. In particular we are grateful to Franco Troiano for his kindness and interest in this activity. We thank Antonello Pasini for the data from the Air Force Meteorological Service. We are grateful to Salvatore Pareti who helped in gathering data used for the validation exercise and to Tiziana Sargolini for the preparation of the manuscript.
